# The enigma of ultraviolet radiation stress granules: Research challenges and new perspectives

**DOI:** 10.3389/fmolb.2022.1066650

**Published:** 2022-12-01

**Authors:** Alexandra J. Cabral, Danielle C. Costello, Natalie G. Farny

**Affiliations:** Department of Biology and Biotechnology, Worcester Polytechnic Institute, Worcester, MA, United States

**Keywords:** stress granules, ultraviolet radiation (UV), biomolecular condensation, poly(A)+ RNA, cell cycle, neurodegeneration

## Abstract

Stress granules (SGs) are non-membrane bound cytoplasmic condensates that form in response to a variety of different stressors. Canonical SGs are thought to have a cytoprotective role, reallocating cellular resources during stress by activation of the integrated stress response (ISR) to inhibit translation and avoid apoptosis. However, different stresses result in compositionally distinct, non-canonical SG formation that is likely pro-apoptotic, though the exact function(s) of both SGs subtypes remain unclear. A unique non-canonical SG subtype is triggered upon exposure to ultraviolet (UV) radiation. While it is generally agreed that UV SGs are *bona fide* SGs due to their dependence upon the core SG nucleating protein Ras GTPase-activating protein-binding protein 1 (G3BP1), the localization of other key components of UV SGs are unknown or under debate. Further, the dynamics of UV SGs are not known, though unique properties such as cell cycle dependence have been observed. This Perspective compiles the available information on SG subtypes and on UV SGs in particular in an attempt to understand the formation, dynamics, and function of these mysterious stress-specific complexes. We identify key gaps in knowledge related to UV SGs, and examine the unique aspects of their formation. We propose that more thorough knowledge of the distinct properties of UV SGs will lead to new avenues of understanding of the function of SGs, as well as their roles in disease.

## Introduction

Stress granules (SGs) are evolutionarily conserved cytoplasmic biomolecular condensates that form in response to a variety of environmental stressors (Nover et al., 1989; Kedersha et al., 1999; Kedersha et al., 2002). Since the discovery of SGs in mammalian cells in 1999 (Kedersha et al., 1999) an ever-broadening array of stressors have been identified as inducers of SGs. While it is known that different stressors can give rise to compositionally and functionally distinct SGs (Aulas et al., 2017; Advani and Ivanov, 2020), our understanding of the components of SG subtypes, and their functional consequences, is understudied. The study of SGs is of intense interest due to the role SGs likely play in various diseases, especially protein aggregation diseases such as Alzheimer’s Disease (AD) and Amyotrophic Lateral Sclerosis (ALS) (Liu-Yesucevitz et al., 2010; Li et al., 2013; Ash et al., 2014; Boyd et al., 2014; Wolozin, 2014; Wolozin and Ivanov, 2019). Because SG function may be determined both by composition and by the environmental conditions under which they form, gaining insights into compositionally distinct SG subtypes will be important for understanding and manipulating SG formation in the context of disease.

One of the most distinct and least understood SG subtypes are those induced by ultraviolet radiation (UV). In countless reports and reviews of SG biology, UV is listed among the litany of stresses that induce SG formation. Humans experience UV daily in the environment, and its genotoxic and carcinogenic effects have been well studied (Roy, 2017). However, published evidence documenting the composition, dynamics, and function of UV-induced SGs is surprisingly limited. We do not yet understand the mechanism(s) driving UV SG assembly, nor the functional consequences of their formation. In this Perspective, we assemble the available evidence on the biology of UV SGs. We believe that this unique SG subtype, which unlike most SGs seems to lack mRNA, is also the only known example of G1-specific cell cycle-controlled SG formation. We propose that the unique properties of UV SGs represent an untapped source for new hypotheses to contribute broadly to the fields of SG biology, aggregation-mediated disease, and biomolecular condensation.

## Understanding stress granule subtypes

### Canonical stress granules

As one of the earliest discovered and most broadly studied SG types, SGs induced by acute high-dose exposure to sodium arsenite have become the canon by which SGs are defined (Kedersha et al., 1999; Kedersha et al., 2002; Aulas and vande Velde, 2015; Aulas et al., 2017; Advani and Ivanov, 2020; Riggs et al., 2020). So-called “canonical” or arsenite-like SGs—caused by many stresses including heat shock (Kedersha et al., 1999), thapsigargin (Aulas et al., 2017), and bisphenol A (Fay et al., 2021) among others—are associated with the inhibition of global protein synthesis and the preferential translation of stress-induced transcripts (Harding et al., 2000a). Bulk translation is inhibited through two main pathways (Advani and Ivanov, 2019): 1) the phosphorylation of the alpha subunit of eukaryotic initiation factor 2 alpha (P-eIF2α), which occurs in mammalian cells *via* one of four serine/threonine kinases: (heme-regulated eIF2α kinase (HRI) (McEwen et al., 2005), protein kinase R (PKR) (Srivastava et al., 1998), PKR-like ER kinase (PERK) (Harding et al., 2000b) and general control nonderepressible 2 (GCN2) (Wek et al., 1995); and 2) the inhibition of the mammalian target of rapamycin (mTOR) (Proud, 2019). The former mechanism inhibits recycling of the eIF2/tRNA_i_^Met^/GTP ternary complex required for translation initiation (Wek, 2018), while the latter mechanism results in hypophosphorylated eIF4E binding protein (4E-BP) which binds eIF4E and inhibits translation (Proud, 2019). When translation is inhibited, 48S translation pre-initiation complexes and untranslated mRNAs accumulate and aggregate in the cytoplasm, favoring a liquid-liquid phase separation (LLPS) event (Guillén-Boixet et al., 2020; Sanders et al., 2020; Yang et al., 2020) and assembling into SGs. Canonical SGs are in dynamic equilibrium with polysomes, as drugs that inhibit polysome disassembly (e.g., emetine, cycloheximide) inhibit SG formation, while drugs that induce premature polysome disassembly (e.g., puromycin) promote SG assembly (Kedersha et al., 2000; Kedersha et al., 2002).

The first protein markers associated with SGs were the RNA-binding proteins TIA-1 and TIAR, as well as the cytoplasmic poly(A)-binding protein (PABPC1) (Kedersha et al., 1999). Soon thereafter, the Ras GTPase-activating protein-binding protein 1 (G3BP1) was identified as a key modulator of SG assembly (Tourrière et al., 2003). It is now firmly established that G3BP1 is a master regulator of SG assembly, and most known SG subtypes require G3BP1 or its close homolog G3BP2 for their assembly (Kedersha et al., 2016; Guillén-Boixet et al., 2020; Sanders et al., 2020; Yang et al., 2020). Related to their relationship with eIF2α-mediated translational control, canonical SGs contain poly(A)+ mRNAs as well as stalled translation 48S-preinitiation complexes including the eIF3 complex, eIF4G, and the small ribosomal subunit (Kedersha et al., 2002; Aulas et al., 2017) (Table 1).

**TABLE 1:** A comparison of key features of canonical, non-canonical, chronic, and pathogenic SG subtypes.

	Acute canonical SG	Acute non-canonical SG			Chronic SG	Pathogenic SGs	
Canonical SG component	Arsenite (As^III^)	UV	Selenite	Nitric oxide (NO)	Chronic nutrient starvation (stSGs)	Tau-associated	ALS-associated
P-eIF2α dependent	✓	✕	✕	✓	✓	N/A	N/A
G3BP1	✓	✓	✓	✓	✓	?	✓
eIF3	✓	✕	✕	✕	✓	✓	✓
Poly(A)+ RNA	✓	✕	✓	✓	✓	✓	✓
RACK1	✓	✕	✕	✓	✕	?	✓
Cell death association	Pro-survival	Pro-death	?	Pro-death	Pro-death	Precedes neuronal cell death	Precedes neuronal cell death
Reference	Aulas et al. (2017)	Moutaoufik et al. (2014), Aulas et al. (2017), Ying and Khaperskyy (2020); this work	Fujimura et al. (2012)	Aulas et al. (2018)	Reineke et al. (2018)	Vanderweyde et al. (2012), Lester et al. (2021)	Gal et al. (2016), Russo et al. (2017), Kamelgarn et al. (2018)

While the composition of canonical SGs have been intensively catalogued through affinity labeling and biochemical purification studies (Jain et al., 2016; Markmiller et al., 2018; Youn et al., 2019; Marmor-Kollet et al., 2020; An et al., 2022), the functional consequences of SG formation remain unclear. One proposed function for SGs is the modulation of apoptosis^12,13^ (Arimoto et al., 2008; Arimoto-Matsuzaki et al., 2016; Park et al., 2020). In a now classic report, Arimoto et al. showed that SGs caused by overexpression of G3BP1 conferred resistance to GADD45-mediated apoptosis (Arimoto et al., 2008). They further demonstrated that hypoxia, which triggers SGs, protected cells from etoposide-mediated apoptosis. In both cases, they confirmed that sequestration of the signaling scaffold protein RACK1 at the SG was essential for protection from apoptosis (Arimoto et al., 2008). Other putative functions for SGs include mRNA triage (Kedersha et al., 2005) and stress-induced translational control (Dey et al., 2010); while SGs likely play a role, the idea that SG formation is absolutely essential or even largely responsible for such processes has largely been discredited (Mateju et al., 2020; Glauninger et al., 2022; Mateju and Chao, 2022).

SGs are dynamic. Canonical SGs rapidly exchange factors with the surrounding cytoplasm, though some factors exchange more rapidly than others (Kedersha et al., 2000). The extent to which factors move in and out of SGs is related to the structure of the granule, which appears to have a less dynamic core surrounded by a more dynamic shell (Wheeler et al., 2016). SGs that are more liquid-like also tend to be more dynamic, whereas those associated with gel or solid-like states (including some non-canonical and pathogenic subtypes, described below) are less dynamic (Kroschwald et al., 2015; Zhang et al., 2019).

### Non-canonical stress granules

The so-called “non-canonical” SG is a category used to collect SGs that do not mirror the formation, composition or function of canonical SGs as primarily understood from studies of arsenite and heat shock SGs (Advani and Ivanov, 2020). This SG subtype was originally dubbed non-canonical based on the lack of the canonical SG protein eukaryotic initiation factor 3 (eIF3) (Advani and Ivanov, 2020; Hofmann et al., 2021) in SGs triggered by sodium selenite (Fujimura et al., 2012), which suggested that the 48S preinitiation complex was not recruited to these SGs. Like canonical SGs, non-canonical SGs form acutely (in 4 h or less of stress), require G3BP1 or G3BP2 for their formation (Yang et al., 2020), and are associated with translational arrest, though they may or may not be associated with eIF2α phosphorylation depending upon the stress (Aulas et al., 2017; Advani and Ivanov, 2020; Hofmann et al., 2021). Non-canonical SGs are reported to form in response to many stresses including sodium selenite (Fujimura et al., 2012), nitric oxide (NO) (Aulas et al., 2018), the eIF4A inhibitor Rocaglamide A (RocA) (Aulas et al., 2017), hydrogen peroxide (Emara et al., 2012), and UV radiation (Kedersha et al., 1999; Pothof et al., 2009). There is broad variation within this non-canonical category as to which components do and do not localize at the SG, examples of which are described in Table 1. Others have attempted to further subdivide canonical and non-canonical SGs into designations including Types I, II, and III (Hofmann et al., 2021), however there is no consensus and some conflicting characterizations among these classifications (Advani and Ivanov, 2020; Hofmann et al., 2021), and thus we will address all acute non-canonical SG subtypes holistically in this Perspective.

Non-canonical SGs are reported to be functionally distinct from canonical SGs in that they are believed to be cytotoxic and less dynamic (Advani and Ivanov, 2020). However, there are few published studies that experimentally validate these characterizations. Relative to cell death, the most thoroughly characterized non-canonical SGs are those caused by nitric oxide (NO). NO SGs were determined to be triggering a non-apoptotic and potentially necrotic cell death by assaying a combination of propidium iodide and trypan blue staining, ATP levels and Caspase-3 cleavage (Aulas et al., 2018). For other non-canonical stresses like selenite, RocA, and UV, a decrease in cell viability was associated with SGs, but a mechanism of death was not elucidated (Fujimura et al., 2012; Aulas et al., 2018). Interestingly, while canonical arsenite-induced SGs are believed to be anti-apoptotic (Arimoto et al., 2008), the inverse relationship does not appear to be a universal feature of non-canonical SG subtypes, in that the lack of sequestration of pro-apoptotic factors like RACK1 at non-canonical SGs cannot simply be interpreted to mean that those pro-apoptotic factors are therefore active and inducing apoptotic cell death associated with non-canonical SG formation. Relative to their dynamics, the only report of which we are aware that directly measured dynamic behavior of a protein component of a non-canonical SG is in the context of NO-induced SGs (Aulas et al., 2018). G3BP1 recovery to photobleached NO SGs was approximately 10% lower than in arsenite SGs (Aulas et al., 2018). To our knowledge, this summarizes the extent to which the cytotoxic and dynamic properties of acute non-canonical SGs have been directly assessed in the literature.

### Chronic, pathological, and membrane-associated stress granule subtypes

The canonical and non-canonical labels are most frequently associated with acute SG formation. Chronic stresses and disease states are associated long-term SG formation. Chronic starvation induced SGs (stSGs) appear after prolonged (8–16 h) starvation of glucose, serum, glutamine and pyruvate (Reineke et al., 2018). These stSGs require both G3BP1 and eIF2α phosphorylation, and are in dynamic equilibrium with polysomes. StGSs appear to be pro-apoptotic, as assessed by Annexin V staining (Reineke et al., 2018), but the dynamic properties of these stSGs remain unknown. Acute metabolic stress, such as complete glucose starvation, glycolysis inhibitors (2-deoxy-D-glucose) and inhibitors of mitochondrial respiration and ATP synthesis (CCCP, oligomycin) are also known to cause stress granules (Kedersha et al., 2002; Wang et al., 2019; Amen and Kaganovich 2020). It is unclear whether or how prolonged metabolic stress relates to acute metabolic stress with respect to SG composition and function, though future investigation in this area could provide a better understanding of the evolution of SGs over prolonged stress conditions.

Pathological SGs (pSGs) are chronic SGs that form in diseased cells and are hypothesized to seed the accumulation of irreversible and toxic disease aggregates. pSGs have been most closely associated to date with ALS and Alzheimer’s Disease (Vanderweyde et al., 2012; Vanderweyde et al., 2016; Ash et al., 2014; Aulas and vande Velde, 2015; Apicco et al., 2018; Zhang et al., 2019; Marmor-Kollet et al., 2020; An et al., 2022). Several excellent reviews have been written on the subject (Wolozin, 2014; Aulas and vande Velde, 2015; Wolozin and Ivanov, 2019), including one within this Research Topic (Rhine et al., 2022). Interestingly, stSGs and pSGs can contain many of the components of canonical granules including poly(A)+ RNA, eIF3, and eIF4G (Table 1), though unlike canonical SGs they are clearly associated with cell death (Liu-Yesucevitz et al., 2010; Vanderweyde et al., 2012; Reineke et al., 2018). This observation raises the question of whether the categorization of SGs into canonical and non-canonical groups based primary on composition data will remain a useful proxy for the breadth of SG function as we continue to discover SGs in new contexts.

Within the past several years, several research groups have noted specific relationships between SGs and membranous organelles (Nicchitta 2022). SGs caused by activation of the unfolded protein response (UPR) can form directly on the endoplasmic reticulum (ER) and incorporate ER-associated mRNAs (Child et al., 2021), which are underrepresented in the transcriptome of SGs caused by arsenite (Khong et al., 2017). These ER-associated SGs contained G3BP1 and were inhibited by cycloheximide, suggesting they are *bona fide* SGs. SGs were also recently reported to associate with the ER in an autophagy-independent disassembly process that was specific for SGs caused by heat shock (Gwon et al., 2021). It is unknown whether SGs caused by other stresses can associate with the ER, however it has been observed that the ER acts as a facilitator of fission of both SGs and processing bodies (PBs) caused by arsenite stress (Lee et al., 2020). Relationships to other membranous organelles have also been noted. SG nucleation localized to the plasma membrane has been observed in yeast in response to starvation, and was linked to protein kinase C signaling under stress (Amen and Kaganovich 2020). Lysosomal damage was found to trigger SG formation, and a subset of those granules were physically associated with the damaged lysosome (Jia et al., 2022). Similarly, significant SG association with the mitochondrial membrane and with peroxisomes has been reported (Amen and Kaganovich 2021). The composition and function of these membrane-associated SGs remains unclear, but their observation adds an interesting dimension to our consideration of the role of SGs in cellular physiology.

## Ultraviolet radiation stress granules are a unique non-canonical stress granule subtype

### Discovery and early observations of ultraviolet radiation stress granules

UV SGs were first reported in 1999 by Kedersha et al. (1999) (as data not shown) as containing the SG marker proteins TIA-1, TIAR, and PABPC1. The first published evidence of UV-induced cytoplasmic aggregation was in 2005, when Teixeira et al. were investigating processing bodies [PBs, a cytoplasmic mRNP complex associated with mRNA decay (Luo et al., 2018)], in the budding yeast *S. cerevisiae* (Teixeira et al., 2005). Then in 2008, UV-induced poly(A)+ RNA-containing bodies were reported in *S. cerevisiae* by Gaillard and Aguilera (2008), but were not thought to be *bona fide* SGs due to the lack of co-localization of putative SG homologs. At that time however, SGs had not yet been discovered in *S. cerevisiae*, and thus core SG markers had not been established in that species.

**FIGURE 1:**
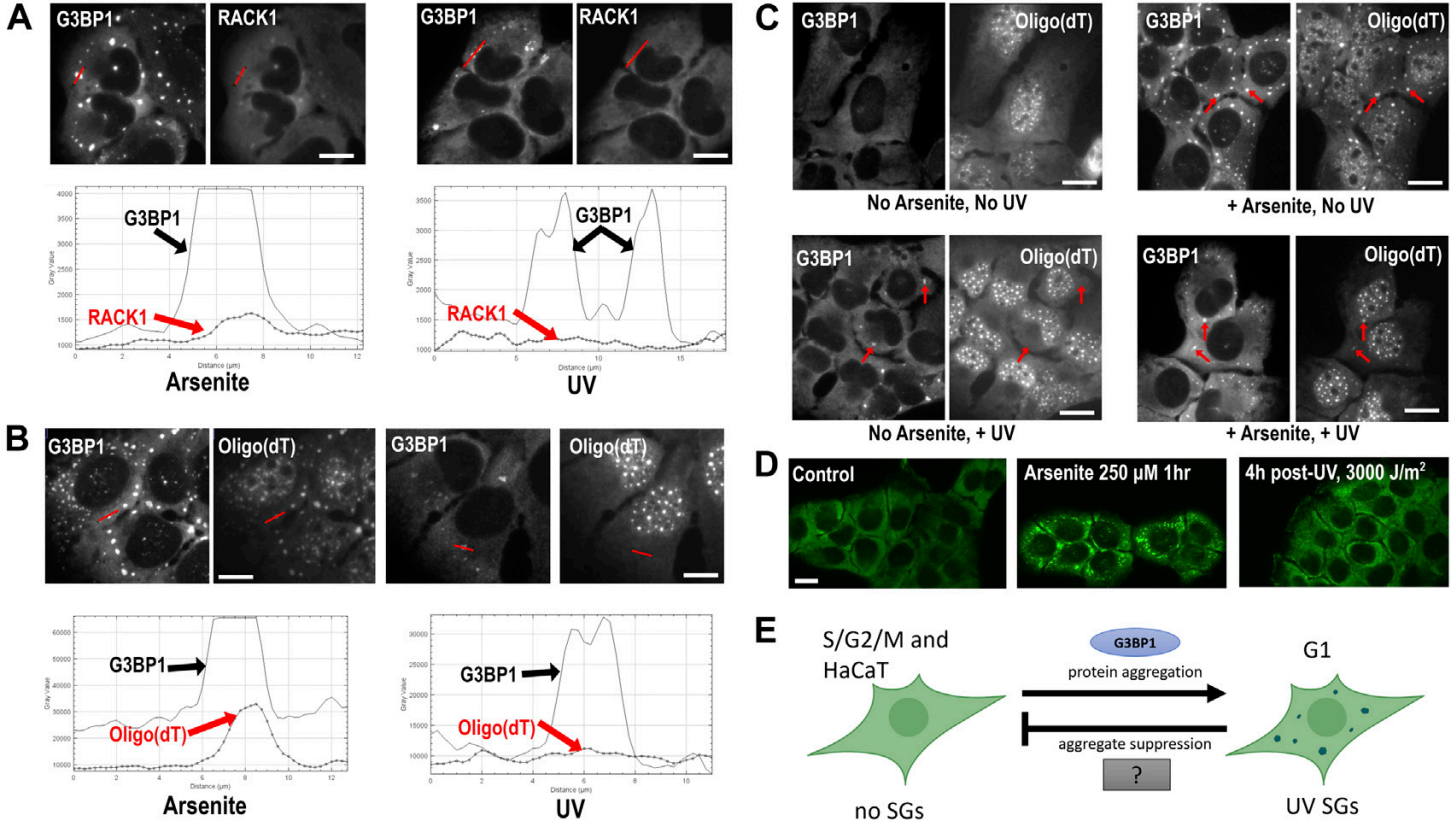
Composition and formation of UV SGs. **(A)** RACK1 does not localize to UV-induced SGs. U2OS treated with arsenite (500 μM, left panels) or UV (15 J/m^2^, then assayed at 4 h post-UV, right panels) and co-stained with antibodies to G3BP1 and RACK1. Profile intensity plots over the red line from each image were compiled using ImageJ. **(B)** U2OS treated with arsenite (500 μM, left panels) or UV (15 J/m^2^, then assayed at 4 h post-UV, right panels) and co-stained with antibodies to G3BP1 and Oligo (dT). Profile intensity plots over the red line from each image were compiled using ImageJ. **(C)** Poly(A)+ RNA SGs can form after UV treatment. U2OS were untreated or treated with UV (15 J/m^2^) for 3 h, then arsenite (500 µM) was added where indicated for a 1 h, then cells were fixed and co-stained with antibodies to G3BP1 and Oligo (dT) FISH. Red arrows point to prominent SGs in each treatment condition. **(D)** HaCaT cells do not form UV SGs. HaCaT cells were exposed to arsenite 250 µM for 1 h, or exposed to 3,000 J/m^2^ UV (4 h post-exposure), then stained with antibody for G3BP1 to assess SG formation. Detailed methods for all panels are available as Supplementary Material online. **(E)** A model for UV SG suppression. See Discussion section for details.

Finally in 2009, Pothof et al. (2009) investigated the DNA damage response (DDR) to UV in HeLa cells, and discovered that the cytoplasmic condensates they observed in response to UV are SGs, based on the co-localization of TIA-1 and Ago2, and that UV SGs may be involved in microRNA-mediated silencing in response to DNA damage. Several more recent publications characterize UV SGs as non-canonical SGs lacking eIF3 and eIF4G (Table 1) (Moutaoufik et al., 2014; Aulas et al., 2017; Ying and Khaperskyy, 2020). We confirm here that UV SGs also lack the pro-apoptotic scaffolding protein RACK1, 4 h after UV treatment of U2OS human osteosarcoma cells (Figure 1A). Like other non-canonical subtypes, UV SGs are G3BP-dependent, eIF2α-independent, and cytotoxic (Pothof et al., 2009; Moutaoufik et al., 2014; Aulas et al., 2017; Ying and Khaperskyy, 2020). Surprisingly however, there are conflicting reports about whether UV SGs contain poly(A)+ RNA (Moutaoufik et al., 2014; Aulas et al., 2017).

### Are ultraviolet radiation stress granules RNA granules?

It was widely assumed in the early years of their discovery that all SGs contained mRNA. SGs are defined as mRNA-containing bodies or mRNP aggregates in many articles and reviews (Anderson and Kedersha, 2006; Riggs et al., 2020; Hofmann et al., 2021). In 2014, Moutaoufik et al. (2014) observed the colocalization of poly(A)+ RNA at UV SGs by co-localizing an oligo (dT) probe with the Fragile X Mental Retardation Protein (FMRP) as a SG marker in NIH-3T3 cells 18 h post-UV exposure. This report is, to our knowledge, the first published account of RNA localization to UV SGs in a mammalian cell type. However, a study published in 2017 by Aulas et al. (2017) found no poly(A)+ RNA localization at UV SGs, using G3BP1 as a SG marker in Hap1 human haploid cells, 2 h post-UV.

To independently validate the observation of poly(A)+ RNA at UV SGs, we treated U2OS cells with 15 J/m^2^ UVC, and used fluorescence *in situ* hybridization (FISH) to examine poly(A)+ RNA localization to UV SGs using G3BP1 as a SG marker. We find, as did Aulas et al. (2017), that poly(A)+ RNA is not enriched at UV SGs at 4 h post-UV exposure, as detectable by FISH (Figure 1B). It is tempting to speculate that perhaps UV light degrades cellular pools of mRNA, which could explain why poly(A)+ RNA FISH signal does not accumulate at UV SGs. However, the literature actually suggests that UV stabilizes and inhibits the degradation of mRNAs (Bollig et al., 2002; Gowrishankar et at., 2005; Gaillard and Aguilera, 2008) Click or tap here to enter text. mRNAs are sequestered in the nucleus upon UV treatment (Burgess et al., 2011) which reduces the cytoplasmic mRNA pool and may contribute to the loss of signal at UV SGs. PABP1 and PABP4 also relocalize to the nucleus, however the poly(A)+ RNA nuclear retention and PABP relocalization under UV stress appear to be independent (Burgess et al., 2011), which may support a model by which PABPs dissociate from their RNA targets under UV stress. Although it is reported UV can stabilize mRNAs, it is also known that UV induces reactive oxygen species and that oxidized RNAs can be targeted for degradation (Wurtmann and Wolin, 2009), meaning that some mRNA degradation may also be possible. Arsenite SGs have been shown to contain reactive oxygen species (ROS), which may further enhance RNA damage and subsequent degradation (Hu et al., 2021), however whether UV SGs contain ROS remains unknown.

To determine whether it was likely that mass degradation of mRNA was responsible for the lack of poly(A)+ RNA we observed at UV SGs, we treated U2OS with UV, then used arsenite to induce SGs at 3 h post-UV exposure. We observe that arsenite SGs induced in UV irradiated cells contain poly(A)+ RNA, though the intensity of localization is decreased compared to arsenite SGs in non-UV treated cells (Figure 1C). Therefore, we submit that mass degradation of mRNA cannot fully explain the lack of poly(A)+ RNA signal at UV SGs, as we show it is possible to assemble poly(A)+-containing SGs after UV treatment. We conclude that poly(A)+ RNAs are either not enriched or not detectable by FISH in UV SGs, though the mechanism of this phenomenonis unknown. As UV SGs are generally smaller than other canonical SGs, it may be the case that the RNA they contain cannot be detected above the cytoplasmic background using FISH. The results do not preclude the possibility that deadenylated mRNAs, non-coding mRNAs and/or small RNAs may still be present at UV SGs, and this possibility warrants further investigation. UV-induced damage may also affect the detection of poly(A)+ RNA using this method. If, however, it is the case that UV SGs are not RNA granules, it would mean that UV SGs do not play any role in the RNA-associated putative functions of SGs such as mRNA triage and translational control.

The finding that UV SGs are depleted of mRNA may also have implications for the assembly of SGs in general. Numerous reports have documented the role of RNA in driving LLPS during SG formation (van Treeck et al., 2018; Guillén-Boixet et al., 2020; Yang et al., 2020; Matheny et al., 2021). If indeed UV SGs do not contain mRNA, then one concludes that the interactions that drive UV SG assembly, unlike most other SG subtypes, must be primarily protein-driven. The lack of facilitation by RNA in LLPS of UV SG formation may also explain why UV SGs have been reported to be smaller and less numerous than other SG subtypes (Moutaoufik et al., 2014). Antiviral ribonucleases generally suppress SGs and have been used to demonstrate the importance of RNAs in SG formation (Burke et al., 2019; Burke et al., 2020); a similar approach could be applied in the case of UV SGs to further confirm or reject a role for RNA in UV SG assembly. Another reasonable prediction is that UV SGs may be more solid or gel-like than canonical or mRNA-containing SGs, as RNA is known to increase the liquid-like properties of biomolecular condensates (Guillén-Boixet et al., 2020; Yang et al., 2020; Bevilacqua et al., 2022). If indeed UV SG assembly is primarily protein-driven, then UV SGs may be a useful model for the specific study of essential protein-protein interactions within SGs. The dynamic properties of UV SGs have not yet been measured, but will no doubt be the topic of future investigation.

### Ultraviolet radiation stress granule suppression

Because SGs have been implicated in seeding pathological protein aggregation (Wolozin and Ivanov, 2019), mechanisms of SG suppression are of intense interest. For example, SG induced by arsenite, a strong oxidizer, can be suppressed by strong antioxidants such as N-acetylcysteine (Szaflarski et al., 2016; Aulas et al., 2018) as well as by preconditioning that likely enhances cellular resilience to oxidative stress (Glass and Wente, 2019; Fay et al., 2021). SGs are also known to be suppressed in mitotic cells, because stalled translation elongation prohibits polysome disassembly in M phase (Gilad et al., 2007). While assessing the DNA damage response, Pothof et al. noted that UV-induced SGs did not form in cells that were positive for cyclin A, a marker of S phase (Pothof et al., 2009). By synchronizing their cells prior to irradiation, it was determined that UV SGs only appeared in cells that were irradiated in G2, passed through mitosis, and were in G1 phase at the assay timepoint. This observation of cell cycle dependence likely explains why only ∼10%–30% of cells in an asynchronous culture form UV SGs (Pothof et al., 2009; Aulas et al., 2017). The mechanism for the G1 specificity of UV SGs is unknown, however the ATM and ATR DNA damage checkpoint kinases were not required for UV SG formation (Pothof et al., 2009).

The observation of cell cycle dependence of UV SGs was confirmed in 2014 by Moutaoufik et al. (2014), who discovered that a dose of 10 J/m^2^ UV resulted in complete G1 arrest. These researchers used FACS profiling to assess the cell cycle, while concurrently assessing SG formation by immunofluorescence microscopy, and reported that G1 arrest correlated with the highest SG formation, and resumption of the cell cycle corresponded with SG clearance. Although this implies UV SGs may be dissolvable, it is also possible that the cells with UV SGs underwent cell death, leaving behind SG-free cells to resume the cell cycle. Thus, suppression of UV SGs in S and G2 phases is a unique feature of UV SGs.

Because skin is the organ most directly affected by UV, we decided to examine UV SG formation in an untransformed human keratinocyte cell line, HaCaT. To our great surprise, we were completely unable to induce UV SGs in HaCaT cells. We applied increasing amounts of UV, from our starting dose of 15 J/M^2^ up to 3,000 J/M^2^, and observed zero UV SG formation in HaCaTs by G3BP1 staining (Figure 1D). We do not yet know if this intriguing observation of UV SG suppression is a common property of all keratinocytes or specific to HaCaT, nor do we know whether it is related to the mechanisms of G2, S, and M phase suppression described above. We should note here that all studies of UV SGs reported to date, including the data reported herein, use UVC (254 nm) light sources. UVC is well known to cause damage to human tissues (Roy, 2017), but does not penetrate the Earth’s atmosphere. It remains unknown whether UVA (400–315 nm) and UVB (315–280 nm) rays can trigger UV SG formation in any cell type, including keratinocytes. However, UVA causes significantly more reactive oxygen species in human tissues than other UV wavelengths (Karran and Brem, 2016), which would cause an increase in cellular stress over other wavelengths.

## Discussion

The observation that UV SGs are RNA-depleted, protein-driven, and cell cycle dependent bodies makes them unique among known SG subtypes, and therefore they are fascinating research targets. Because UV SGs do not appear to contain much if any RNA (Figure 1B), we hypothesize that they primarily represent the effects of protein-protein interaction-driven condensation. In this way, UV SGs are a platform for studying the protein-specific drivers and suppressors of SG phase separation. The observations of SG suppression in S/G2/M phases, as well as in keratinocytes (Figure 1D), coupled with the apparent lack of poly(A)+ RNA (Figure 1B), informs our model for UV SG suppression (Figure 1E). In response to UV, which may cause nuclear retention and/or RNA-binding protein dissociation from RNA (Burgess et al., 2011), the protein drivers of SG formation including G3BP1/2 are just barely capable of surpassing a critical threshold and achieving protein-driven phase separation. In S/G2/M phases, and in certain UV-prone cell types such as keratinocytes, yet unknown cytoplasmic factors suppress phase separation by shifting the equilibrium to disfavor protein-driven condensation. This model is supported by the observation that G3BP1 condensation is significantly facilitated by the presence of RNA both *in vitro* and *in vivo* (Guillén-Boixet et al., 2020; Yang et al., 2020). Thus, in the absence of RNA, cytoplasmic factors may play a greater role in favoring or disfavoring biomolecular condensation in a way that would not be detectable if RNA were there to push the balance toward condensate formation. The “mystery SG suppressor” may not be a single gene, but a set of cytoplasmic environmental conditions associated with cell cycle progression. Cross-referencing proteomic, transcriptomic, and metabolomic differences between G1 and the rest of the cell cycle with similar data from keratinocytes is a starting point to identify the hidden factor(s) regulating UV SG suppression.

The unique nature of UV SGs may have revealed to us a path forward to identify suppressors of other protein aggregates. Many reviews have built powerful arguments for the relationship of SGs to protein aggregation diseases including ALS and AD (Li et al., 2013; Ash et al., 2014; Wolozin, 2014; Aulas and vande Velde, 2015; Wolozin and Ivanov, 2019). We suggest that the identification of UV SG suppressors, as modulators of a protein-driven condensation process, represent a new Frontier for research in neurodegeneration. If SGs can become pathological and can seed toxic aggregation of proteins like tau and TDP-34, then suppressors of SG formation may prevent or even reverse these seeds and thereby prevent disease aggregates from forming. It is also worth noting that the affected cells in neurodegenerative disease—neurons—are perpetually frozen in G0; UV SGs are repressed in S/G2/M phases. If we can understand the cellular conditions underlying cell cycle-dependent UV SG suppression, we could potentially apply this knowledge to create a G2-like state in neurons and thereby suppress pathological aggregates. In light of their potential to illuminate new paths for protein aggregation disease research, addressing these questions about an oft-neglected SG subtype takes on a fresh urgency.

Many key gaps in knowledge about non-canonical SGs in general, and UV SGs in particular, remain to be addressed. It is still entirely unclear how UV irradiation triggers SG formation in the first place. The lack of poly(A)+ RNA suggests that perhaps G3BP1, and other RNA-binding proteins like PABPs (Burgess et al., 2011) may dissociate from their mRNA targets in response to UV stress. But the pathways that would lead to such dissociation are unknown. Identifying the components of UV SGs is a key step to understanding these unusual SGs. Biochemical purification strategies and proximity labeling studies have enabled us to understand the proteomic and transcriptomic landscapes of canonical SGs (Jain et al., 2016; Khong et al., 2017; Markmiller et al., 2018; Namkoong et al., 2018; Padrón et al., 2019; Youn et al., 2019; Marmor-Kollet et al., 2020; Matheny et al., 2021; Vu et al., 2021; An et al., 2022). However, parallel studies on UV SGs, or any non-canonical subtype, remain to be performed. The dynamics of UV SGs have not been directly measured, and while the evidence presented here leads us to predict that UV SGs have a more solid or gel-like than liquid like behavior, for now their dynamic nature is unclear. It further has not been investigated whether UV SGs, like canonical subtypes, are also organized into core and shell subdomains, or whether their substructure is substantially distinct form known SGs. Finally, while we have not addressed the topic here, most canonical and non-canonical SGs are believed to be reversible and to dissolve upon the resolution of stress, facilitated by the autophagy pathway (Buchan et al., 2013). The mechanisms of UV SG dissolution, if they are in fact reversible, remain unknown.

## Data Availability

The raw data supporting the conclusion of this article will be made available by the authors, without undue reservation.
